# The life expectancy gap: Socioeconomic differences in life expectancy between areas in Germany

**DOI:** 10.25646/13026

**Published:** 2025-03-17

**Authors:** Jens Hoebel, Niels Michalski, Jens Baumert, Enno Nowossadeck, Fabian Tetzlaff

**Affiliations:** Robert Koch Institute, Department of Epidemiology and Health Monitoring, Berlin, Germany

**Keywords:** Social determinants, Health inequality, Life expectancy, Mortality, Regional deprivation

## Abstract

**Background:**

This study examines differences in life expectancy between Germany’s most affluent and most deprived areas.

**Methods:**

Nationwide data from the cause-of-death statistics from 2003 to 2022 were linked with official population data to calculate the average life expectancy of females and males in each of Germany’s districts. Regression analysis was used to assess the association with the German Index of Socioeconomic Deprivation (GISD) at district level and calculate the life expectancy gap between the most and least deprived areas.

**Results:**

In the period 2020 – 2022, life expectancy in the most deprived areas was 4.3 years (females) and 7.2 years (males) lower than in the least deprived areas. In the period 2003 – 2005, this life expectancy gap was still 2.6 and 5.7 years. The widening of the life expectancy gap resulted from a less favourable development of life expectancy in the most deprived areas. It was already evident before and intensified during the COVID-19 pandemic.

**Conclusions:**

The increasing life expectancy gap indicates that health inequality in Germany is increasing. As a result, the development of a strategy to improve health equity is more important than ever to be placed on the policy agenda.

## 1. Introduction

Numerous studies have shown a close relationship between socioeconomic position and health in the population [1–3]. Even in a high-income country like Germany, people in socioeconomically disadvantaged circumstances have poorer health and higher risks of disease than those in more advantaged socioeconomic circumstances [4, 5]. As an extreme manifestation, health inequality is reflected in earlier death and shorter life expectancy of people in socioeconomically disadvantaged groups [6, 7].

Life expectancy is an important summary measure of the health status of a population. It indicates how many years a newborn can expect to live on average if mortality rates during a given period remain constant throughout his or her lifetime [8, 9]. Differences in average life expectancy between socioeconomically disadvantaged and privileged groups can be understood as a global measure of the degree of health equity within a country.

On the one hand, socioeconomic mortality differentials within a country can be observed between groups of individuals, e.g. between individuals with low and high income [6, 10, 11]. On the other hand, they can also be identified at the area level, i.e. between the local populations of socioeconomically disadvantaged and affluent areas of a country, as is the case in Germany [12–14]. For Germany as a whole, data from 2019 show that women and men residing in the socioeconomically most disadvantaged fifth of areas have a 33 % and 43 % higher risk of premature death, respectively, than their peers in the most affluent fifth of areas [13]. This is reflected in the shorter life expectancy of females and males in disadvantaged areas [12, 15]. Findings on area-level socioeconomic differences in mortality not only allow conclusions to be drawn about the degree of health equity, but also about the equivalence of living conditions in Germany [16]. The goal of ‘establishing equivalent living conditions throughout the federal territory’ has been enshrined in Article 72 of the German Basic Law, Germany’s constitution, since 1994.

This paper presents calculations of the socioeconomic ‘life expectancy gap’ between Germany’s areas, i.e. the difference in life expectancy between the most and least deprived areas in the country. In order to draw conclusions about the development of social inequality in life expectancy across Germany, both current findings and time trends of this life expectancy gap since the beginning of the 2000s are presented.

## 2. Methods

### 2.1 Data

The analysis is based on data from the German official cause-of-death statistics [17], official population updates and German Index of Socioeconomic Deprivation (GISD) [15] for the period from 2003 to 2022. The cause-of-death statistics contain information on all officially registered deaths of persons with permanent residence in Germany. In addition to the dates of birth and death, sex and cause of death, place of residence is available in the data. This allows the data to be analysed at a small-area level and linked with other area-based data in compliance with data protection regulations and confidentiality rules. The linkage was conducted at the level of Germany’s 400 districts. The life expectancy values for the whole of Germany stem from the information system of Germany’s federal health reporting [18]. To calculate district-specific life expectancies in the present study, the microdata from the cause-of-death statistics were analysed at the Office for Statistics Berlin-Brandenburg’s on-site workstations for guest researchers. To calculate the life expectancy gap (see calculation method below), this life expectancy data was then linked at the Robert Koch Institute with the district-specific GISD value of the intermediate year in a 3-year period. Due to the higher life expectancy of women, the calculations were conducted separately for females and males. The analysis was performed using Stata 17.0 SE (StataCorp LLC, College Station, TX, USA).

**Key messages**

► Women and men in Germany’s most deprived areas have a significantly shorter life expectancy than those in the most affluent areas.
► In the 2000s and from the mid-2010s onwards, the life expectancy gap between Germany’s affluent and deprived areas widened.
► The widening of the life expectancy gap was exacerbated during the COVID-19 pandemic.
► Life expectancy has developed less favourably in deprived than in affluent areas.

### 2.2 Area-level socioeconomic deprivation

To determine the socioeconomic situation of the deceased’s area of residence, the GISD [15, 19] was used at the level of Germany’s 400 districts (GISD Release 2025 v1.0). GISD is a measure of relative socioeconomic deprivation in areas of Germany and is composed of nine spatially aggregated single indicators, which represent the three core dimensions of socioeconomic inequality (education, employment, income). Each dimension is represented by three single indicators. Examples are the proportion of school dropouts without certificate, the unemployment rate or the average disposable household income in the areas. The single indicators are weighted in their dimension by their factor loadings, which are derived from a principal component analysis. Subsequently, the three dimensions of education, employment and income are equally weighted in the total index. After normalisation, the GISD ranges from 0 (least deprived) to 1 (most deprived) [15].

### 2.3 Calculation of the life expectancy gap

To calculate the life expectancy gap between the most and least deprived areas, we first calculated average life expectancy at birth (in years) for each district using life tables [8]. The life tables were calculated using combined 5-year age groups and in moving 3-year periods in order to minimise random fluctuations in small districts with small numbers of deaths. Using linear regression, the life expectancy values of the districts were then regressed on the districts’ GISD values. As the GISD varies from 0 to 1, the regression coefficient indicates by how many years life expectancy differs on average between the most and least deprived districts. In each 3-year period, the GISD extreme values of 0 and 1 were each represented by one district.

**Figure 1:**
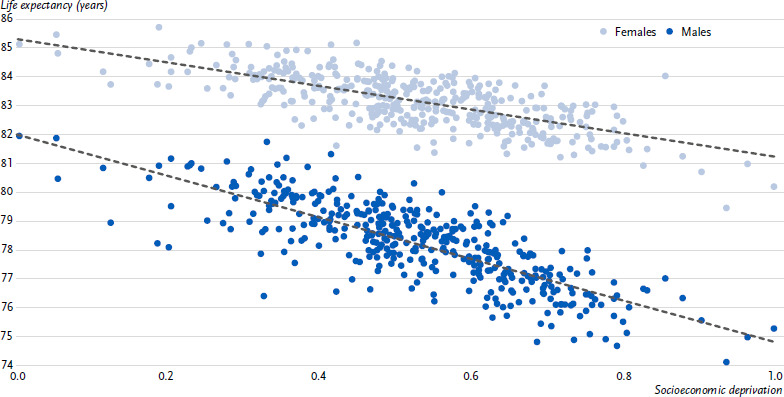
Average life expectancy in districts by sex and area-level socioeconomic deprivation, 2020 – 2022. Explanation of the x-axis: 0 = lowest deprivation, 1 = highest deprivation. Source: Own calculations based on cause-of-death statistics [17], population updates and the German Index of Socioeconomic Deprivation (GISD) [15, 19]

Using the regression-based method, the life expectancy and deprivation values of all districts were included in the calculation of this difference (Figure 1). This way, the inequality of life expectancy was considered across the entire range of district-level socioeconomic deprivation in Germany and summarised in one figure. As an extreme comparison, this value reflects the life expectancy gap between the most and least deprived areas per 3-year period, which corresponds to the distance of the y-values (life expectancy) between the highest and lowest x-values (socioeconomic deprivation) on the fitting line in Figure 1. The regression-based calculation also contributes to the robustness of the ‘life expectancy gap’ indicator, as the inclusion of values from all districts compensates for random fluctuations in single, e.g. low-population districts.

In addition, the question of whether changes in the life expectancy gap were due to changes in life expectancy in the most and/or least deprived areas was investigated. For this purpose, the life expectancy values for areas with GISD = 0 and areas with GISD = 1 were estimated from the above-mentioned regression, separately for each 3-year period.

## 3. Results

In the first 3-year period from 2003 to 2005, average life expectancy was 81.8 years for females and 76.2 years for males. Over the period considered, life expectancy increased to a total of 83.2 years for females and 78.3 years for males in 2020 to 2022.

According to the calculation of the life expectancy gap described above, between 2020 and 2022, females in the most deprived areas had a life expectancy that was 4.3 years shorter than among females in the most affluent areas, i.e. those in the least deprived areas. For males, this life expectancy gap was 7.2 years. In the 2000s, the life expectancy gap widened from initially 2.6 years for females and 5.7 years for males in 2003 to 2005 to 3.3 and 6.3 years in 2009 to 2011, respectively (Table 1). While the life expectancy gap slightly narrowed at the beginning of the 2010s, a renewed widening was observed from the mid-2010s onwards. The widening intensified in the period of the COVID-19 pandemic from 2020, so that the highest values for the life expectancy gap were observed at the end of the 20-year period under consideration. Over the entire period from 2003 to 2022, the life expectancy gap was considerably larger among males than females.

**Figure 2:**
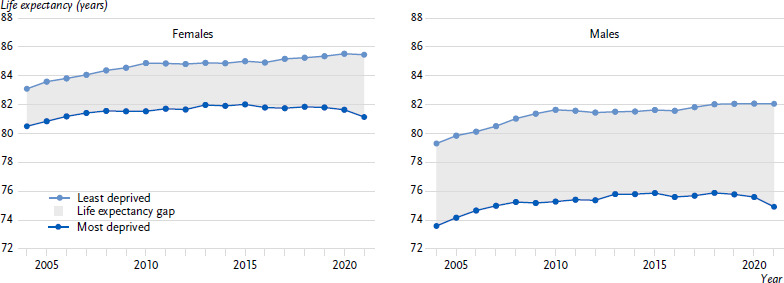
Average life expectancy in Germany’s most and least deprived areas, 2003 – 2022. Source: Own calculations based on cause-of-death statistics [17], population updates and the German Index of Socioeconomic Deprivation (GISD) [15, 19]

The distance in average life expectancy between the most and least deprived areas shown in Figure 2 corresponds to the life expectancy gap. The widening of the life expectancy gap in the 2000s occurred because the most deprived areas benefited less from the general life expectancy gain than the least deprived areas. The renewed widening of the life expectancy gap from the mid-2010s onwards resulted from the fact that life expectancy largely stagnated in the most deprived areas, whereas it further increased in the least deprived areas (Figure 2). During the COVID-19 pandemic, life expectancy declined primarily in highly deprived areas, resulting in the further widening of the life expectancy gap from 2020 onwards.

## 4. Discussion

The results show considerable differences in life expectancy across Germany to the disadvantage of women and men living in socioeconomically disadvantaged areas. As life expectancy has developed more favourably in the most affluent compared to the most deprived areas, the socioeconomic life expectancy gap between areas has widened in recent decades. This widening of the life expectancy gap was exacerbated during the COVID-19 pandemic by a significant decline in life expectancy in areas of high socioeconomic deprivation.

Empirical research into socioeconomic inequalities in life expectancy and their change over time provides important insights into how health equity develops within a country. It is therefore an essential component of national public health surveillance, especially since improving health equity and reducing health inequalities are declared core objectives of public health and part of the Essential Public Health Operations defined by the World Health Organization [20, 21].

The widening of socioeconomic inequalities in life expectancy over the last decades, as indicated by the present findings, has already been visible in previous studies from Germany [10, 22] and has also been reported for some other western countries such as the United States, Norway or Denmark [23–25]. It is internationally assumed that a variety of factors at the societal level can influence the development of health inequalities within a country, including, for example, economic developments, social change, austerity policies and associated reorganisations of the welfare state, labour market, tax system and public services, access to health services or taxation of health-endangering products [26, 27]. During the COVID-19 pandemic, the widening of the life expectancy gap in Germany intensified because COVID-19-related mortality was highest in deprived areas [12, 28], and aspects of infection control may also have played a role. Further research is needed to examine which social, economic, political or, possibly, medical developments in Germany contributed to the widening of the socioeconomic life expectancy gap in recent decades and the relative importance each of these factors had in this process.

However, the possibilities for research into socioeconomic inequalities in life expectancy are considerably more limited in Germany than in many other high-income countries because there are no national mortality data available in Germany that contain individual-level socioeconomic information. This is mainly due to the fact that the German census does not include a mortality follow-up, that the death certificates do not record socioeconomic characteristics of the deceased, and that there is no nationwide mortality register that could be linked to individual socioeconomic information [29, 30]. Thus, other data and methods must be used in Germany. In addition to long-term follow-ups of population samples [6, 31] and routine data from the social insurances [10, 11, 32], ecological study designs can partially bridge this information gap in Germany. Correlational studies at the area level, such as those conducted in the present article, belong to this type of study.

Moreover, the investigation of socioeconomic inequalities in life expectancy at the area level also has an independent value of knowledge, which results from its reference to the goal of ‘establishing equivalent living conditions throughout the federal territory’ declared in the German Basic Law (Art. 72 para. 2 GG). On the one hand, the results can provide information on the extent to which this goal is being approached with regard to health and longevity. On the other hand, they also highlight the fact that the establishment of equivalent living conditions across Germany is also an important field of action for pursuing the public health objective of reducing health inequalities [13].

The finding that socioeconomic differences in mortality and life expectancy are larger among males than females, which is also shown by the presented results on the life expectancy gap between deprived and affluent areas, have already been reported in previous studies [6, 12, 31]. The reasons for this difference might be diverse. Behavioural factors, i.e. characteristics of a healthy or unhealthy lifestyle, may only play a minor or partial role in this respect. Although certain behavioural risk factors, such as smoking or unhealthy diets, are more prevalent among men than women [33, 34], there is no consistent pattern according to which socioeconomic inequality in the prevalence of behavioural risk factors would generally be larger in men than in women [34–36]. Structural and material factors, such as labour force participation and working conditions, may play a more important role in explaining the gender difference in the socioeconomic life expectancy gap, especially since these factors generally make a larger contribution to the socioeconomic gradient in health than behavioural factors [37–40]. Methodological aspects regarding the choice of socioeconomic indicators and the measurement of socioeconomic characteristics in empirical studies could also contribute to finding different levels of mortality inequalities between women and men [41].

### 4.1 Methodological strengths and limitations

Using data from the official cause-of-death statistics, complete data of all deaths in Germany could be used to calculate the life expectancy gap. Compared to sample-based calculations, this has the advantage that the results are not subject to statistical uncertainties that may arise from estimation procedures to extrapolate to the population and from the quality of the sample (e.g. selection bias). However, the calculation of the life expectancy gap at the area level (ecological study design) is subject to limitations. In particular, the possibility of ecological fallacy cannot be ruled out. An ecological fallacy can occur in studies with this design when an empirically identified association between two characteristics at the aggregate level is transferred to the individual level by way of interpretation, although the association does not exist at the individual level. Accordingly, the informative value of the present analysis is limited to the description of spatial inequalities and the identification of disadvantaged areas. Conclusions about causal effects and relationships at the individual level cannot be drawn [15].

Furthermore, it should be noted that in many studies and analyses, areas are grouped into quintiles according to their GISD value, i.e. into fifths of areas with similarly high or low levels of deprivation. In doing so, it is not the full range and variation of the GISD values that are used like in the metric variable. This classification is done, for example, to compare incidence and mortality rates or life expectancies between high and low deprivation quintiles [12, 13, 42, 43]. For the region-based calculation of the life expectancy gap presented in our study, however, the GISD was used metrically in its full resolution. Instead of grouping fifths of areas, the life expectancy in the least and most deprived areas was compared. This resulted in a larger life expectancy gap than in comparisons of quintiles [12].

The regression-based calculation of the life expectancy gap followed an approach analogous to that used in other countries. In the United Kingdom, the life expectancy gap between the most and least deprived areas is quantified by the Office for Health Improvement & Disparities using the Slope Index of Inequality (SII) [44]. The SII is also a regression-based summary measure of the difference in a health outcome (here: life expectancy) between the most and least deprived groups or areas of a country, calculated by taking account of the entire distribution and range of a socioeconomic variable (here: deprivation index) [45, 46]. However, due to differences between countries in spatial granularity and size of areas as well as methods for calculating area-based deprivation indices, the results cannot be compared directly [47].

In order to minimise statistical uncertainties and random fluctuations in small districts with low death counts when calculating the district-specific life expectancies, the life tables were calculated with collapsed age groups and for a cumulative period of three calendar years. In the literature, there are also approaches that use estimation techniques for this purpose [48, 49], and their results may differ from the approach used here, which should be considered when interpreting and comparing the results.

### 4.2 Measures and conclusions

The present results confirm findings according to which health inequalities in Germany have increased in recent decades and health and life chances between socioeconomically disadvantaged and affluent areas are diverging [12, 13, 50]. In view of the public health objectives mentioned above and the goal of establishing equivalent living conditions across Germany enshrined in the German Basic Law, the development of a policy strategy and initiation of suitable measures to improve health equity in Germany is more important than ever to be placed on the policy agenda.

To this end, it would first be important to look at the mechanisms underlying the emergence and reproduction of health inequalities. Structural factors such as working, living and environmental conditions, psychosocial factors such as worries about the future and chronic stress as well as behavioural factors such as an unhealthy lifestyle can be referred to in this regard [51, 52]. Many of these factors influence the risk of diseases that contribute to social differences in premature mortality and life expectancy. These include, for example, cardiovascular diseases, certain cancers or chronic respiratory diseases [12, 13]. In addition, ill-health can make social upward mobility more difficult and lead to social downward mobility [53]. Accordingly, strategies to reduce health inequalities and improve health equity must be multi-facetted. Measures that are primarily aimed at individual behaviour and rely, for example, on providing information or incentives for healthy behaviours are unlikely to be suitable for reducing health inequalities in the long term on their own. This is because such measures generally have no or only short-term effects in disadvantaged socioeconomic groups [54]. Preventive services offered by the healthcare system, such as early detection examinations or incentive programmes offered by statutory health insurance funds, are also more likely to attract members of higher socioeconomic groups and to be taken up by them [55–57]. Such measures therefore run the risk of increasing health inequalities instead of reducing them [58, 59].

Measures aimed at structural changes in the living conditions and environment of socioeconomically disadvantaged people have been found to be more effective in reducing health inequalities [54, 58, 60–62]. This refers to the wider context as well as local conditions in which people grow up, work, live and age. In addition to the legal framework such as regulations on occupational safety, consumer protection, pollution control and health protection, these can be, for example, setting- and community-based interventions for health promotion and prevention at local level or fiscal and social policies at societal level.

An empirical example with reference to regional health inequalities in Germany are increases in social security benefits following German reunification. These were associated with increases in life expectancy, particularly in the socioeconomically less developed areas of eastern Germany [63]. In order to achieve further progress, interventions that combine effective measures at multiple levels (society, community, individual) may be particularly promising for reducing health inequalities [64, 65]. Complex multi-level interventions to reduce health inequalities are still lacking in Germany. In other countries, such as the United Kingdom or Norway [66–68], national action programmes to reduce health inequalities have already been developed and implemented. These programmes involve various levels of action, focus on priority public health issues and were supported by several ministries in the sense of a ‘Health in all Policies’ approach. For the English strategy to reduce health inequalities, which was implemented between 1999 and 2010, reductions in mortality inequalities between England’s deprived and affluent areas could indeed be identified [69, 70].

These experiences suggest that a comprehensive and cross-policy action strategy can achieve success in reducing health inequalities. In addition, not only various policy departments should be involved, but also a large number of stakeholders from research, practice, politics and civil society. People from socially disadvantaged groups should also be actively involved in order to be able to incorporate their concerns and perspectives and develop effective measures.

## Figures and Tables

**Table 1:** Life expectancy gap (in years) between Germany’s most and least deprived areas across moving 3-year periods, 2003 – 2022. Source: Own calculations based on cause-of-death statistics [17], population updates and the German Index of Socioeconomic Deprivation (GISD) [15, 19]

	Females (years)	Males (years)
2003 – 2005	2.6	5.7
2004 – 2006	2.8	5.8
2005 – 2007	2.6	5.5
2006 – 2008	2.6	5.6
2007 – 2009	2.8	5.8
2008 – 2010	3.0	6.2
2009 – 2011	3.3	6.3
2010 – 2012	3.1	6.1
2011 – 2013	3.0	6.0
2012 – 2014	2.9	5.7
2013 – 2015	2.8	5.7
2014 – 2016	2.9	5.8
2015 – 2017	3.0	5.9
2016 – 2018	3.3	6.1
2017 – 2019	3.3	6.2
2018 – 2020	3.5	6.4
2019 – 2021	3.8	6.6
2020 – 2022	4.3	7.2
